# Submicroscopic carriage of *Plasmodium falciparum* and *Plasmodium vivax* in a low endemic area in Ethiopia where no parasitaemia was detected by microscopy or rapid diagnostic test

**DOI:** 10.1186/s12936-015-0821-1

**Published:** 2015-08-05

**Authors:** Fitsum G Tadesse, Helmi Pett, Amrish Baidjoe, Kjerstin Lanke, Lynn Grignard, Colin Sutherland, Tom Hall, Chris Drakeley, Teun Bousema, Hassen Mamo

**Affiliations:** Department of Medical Microbiology, Radboud University Medical Centre, Geert Grooteplein 26-28, 6525GA Nijmegen, The Netherlands; Medical Biotechnology Unit, Institute of Biotechnology, Addis Ababa University, POBox 1176, Addis Ababa, Ethiopia; Department of Infectious and Tropical Diseases, London School of Hygiene and Tropical Medicine, Keppel Street, London, WC1E 7HT UK; Department of Microbial, Cellular and Molecular Biology, College of Natural Sciences, Addis Ababa University, POBox 1176, Addis Ababa, Ethiopia

**Keywords:** Malaria, Asymptomatic, Submicroscopic, *Plasmodium falciparum*, *Plasmodium vivax*, Elimination, G6PD deficiency, 8-aminoquinolines

## Abstract

**Background:**

Motivated by the success in malaria control that was documented over the last decade Ethiopia is aiming at malaria elimination by 2020 in selected districts. It is currently unknown if asymptomatic, submicroscopic malaria parasite carriage may form a hurdle to achieve elimination. The elimination effort may further be complicated by possible glucose-6 phosphate dehydrogenase (G6PD) deficiency which would hinder the use of 8-aminoquinolines in the elimination efforts.

**Method:**

In February 2014 a community-based cross-sectional survey was conducted in Malo, southwest Ethiopia. Finger-prick blood samples (n = 555) were tested for presence of *Plasmodium falciparum* and *Plasmodium vivax* with microscopy, rapid diagnostic test (RDT), and nested polymerase chain reaction (nPCR). Multiplicity of *P. falciparum* infections was determined based on genotyping the polymorphic merozoite surface protein-2 (*MSP-2*) gene. Individuals were also genotyped for mutations in the gene that produces G6PD.

**Results:**

All study participants were malaria infection negative by microscopy and RDT. Nested PCR revealed *P. falciparum* mono-infection in 5.2% (29/555), *P. vivax* mono-infection in 4.3% (24/555) and mixed infection in 0.2% (1/555) of individuals. All parasitemic individuals were afebrile (axillary temperature <37.5°C). None of the study participants carried mutations for the *G6PD* African A-(202GA) and Mediterranean (563CT) variants. All infections, except one, were single-clone infection by *MSP-2 genotyping*.

**Conclusion:**

The detection of a substantial number of subpatent malaria infections in an apparently asymptomatic population without evidence for malaria transmission by conventional diagnostics raises questions about the path to malaria elimination. It is currently unknown how important these infections are for sustaining malaria transmission in the study sites. The absence of G6PD deficiency indicates that 8-aminoquinolines may be safely deployed to accelerate elimination initiatives.

## Background

Over the last decade, several malaria-endemic countries have made major progress in their fight against malaria. Between 2000 and 2013 estimated malaria mortality rates fell by 47% worldwide and by 54% in sub-Saharan Africa [1]. In Ethiopia confirmed malaria cases declined by 66% in 2011 compared to the pre-intervention period in 2001–2005 [2]. This success has mostly been attributed to the scale-up of conventional malaria control interventions such as widespread availability of insecticide-treated nets, indoor residual spraying and the availability of artemisinin combination therapy.

Encouraged by the remarkable success, several malaria-endemic countries have adopted elimination strategies [3]. To achieve successful malaria elimination, strategies have to consider all malaria-infected individuals for interventions. Populations of interest for malaria elimination efforts include asymptomatic infections since these can sustain an ongoing malaria transmission [4]. In settings where recent malaria control efforts have been successful, low density submicroscopic infections are particularly prevalent [5]. There is debate about the relative importance of these submicroscopic infections for onward malaria transmission [4], although current evidence indicates that these low density infections need to be considered in strategies that aim at malaria elimination and require diagnostics that are more sensitive than microscopy and rapid diagnostic test (RDT) [6].

In its current national malaria strategic plan (2014–2020), Ethiopia declared to achieve elimination in low malaria transmission settings by the end of 2020 [7]. Three quarter of the country, where 68% of the total population is living in, is malarious [8]. The transmission pattern of the disease is highly heterogeneous in space and time owing to variation in altitude and rainfall. Recent reports indicated that most part of the country has low malaria transmission [9–11]. However, the magnitude of asymptomatic and submicroscopic infections has not yet been studied well and may form a stumbling block to achieve the set goals.

The aim of this study was to assess the degree of asymptomatic and submicroscopic malaria parasite carriage in two low endemic settings of Malo-Koza district in southwest Ethiopia. To support future policy considerations that may involve treatment with primaquine for both *Plasmodium falciparum* gametocytes and *Plasmodium vivax* liver-stages [12], glucose 6-phosphate dehydrogenase (*G6PD)* genotype was also assessed. Primaquine administration in *G6PD* deficient individuals is associated with a dose-dependent risk of 8-aminoquinolines-induced haemolysis that could be life-threatening in specific cases [13].

## Methods

### Study sites

The study was conducted in Malo (6°26′0″ N latitude and 36°38′0″ E longitude), a mountainous area in southwest Ethiopia that is located 650 km from the capital, Addis Ababa. Inhabitants of Malo occupy the middle Omo river basin. The study was conducted specifically in two villages: Salayish Mender 4 (SM4) and Tatta-qirchiqircho (TQ). Malaria transmission in both sites is seasonal with peaks in transmission in September to mid-November and April to May following the major (*kiremt*) and minor (*belg*) rainy seasons, respectively.

SM4, which is a government-sponsored settlement area, is located at an elevation of c 1,100–1,300 m and constitutes ethnically and culturally heterogeneous population groups. The households are mostly designed with grass thatch roof, bamboo walls (sometimes wood or mud) and earth floor without cement; the floor and wall are plastered with animal dung. TQ is a midland area situated in the escarpments of two perennial rivers (Mitsilito and Tullo rivers).

### Ethical considerations

Project approval was granted by the Institutional Research Ethics Review Board of the College of Natural Sciences, Addis Ababa University. Prior to sample collection informed written consent was obtained from adult participants and parents/legal guardians for children below the age of 18 years.

### Sample collection

This community-based survey was conducted in February 2014, in the dry season. Community members residing in the study sites for at least 2 years were invited to participate and members who gave their informed written consent were included in the study. No formal sample size calculation was performed and the objective was to sample the largest possible fraction of the population. Finger-prick blood samples were collected for malaria microscopy, RDT (First Response^®^ malaria Antigen pLDH/HRP2 *P.f.* and Pan Combo Card Test; Premier Medical Corporation Ltd, Dist. Valsad, India) and dried blood spots on Whatman 3MM filter papers (Whatman, Maidstone, UK). Thick and thin blood smears were prepared, Giemsa-stained and microscopically scanned for malaria parasites. A slide was declared malaria-negative when *Plasmodium* was not detected in 100 high power fields examined by oil immersion (100×); all slides were read by two independent microscopists with a third reader being consulted in case of discordant results. Body temperature was measured for all individuals. Those with an axillary temperature ≥37.5°C and a positive blood slide or RDT were defined as symptomatic malaria patients.

### DNA extraction and parasite genotyping

DNA was extracted using Saponin-Chelex extraction as previously described [14] from two punches of 2.5 mm diameter. DNA was eluted in 100 µL of a 6% Chelex in DNase/RNase free water solution and stored at −20°C until further use. Nested polymerase chain reaction (nPCR) assays were performed to detect the presence of the small ribosomal subunit (18S) of *P. falciparum* and *P. vivax* [15]. The limit of detection of this method was ≥1 parasite/µl of blood in our laboratory, estimated based on serial dilutions of cultured NF54 parasites (Baidjoe, unpublished observations). Pooled DNA isolates from *P. falciparum* NF54 cultures (Radboudumc, Nijmegen, The Netherlands) and *P. vivax* Malaria Reference Laboratory positive control (London School of Hygiene and Tropical Medicine, London, UK) were included on every PCR plate as positive controls, alongside a negative water sample control. Samples were visualized on a 2% agarose ethidium bromide gel by electrophoreses and results were subsequently visualized on UV-imager.

For all 18S *P. falciparum*-positive samples, the complexity of infection was determined based on the polymorphic merozoite surface protein-2 (*MSP-2*) [16]. In brief, 5 µl of DNA was added to a primary master mix and run in 50 µl final volume. Subsequently, 1 µl of the primary PCR product was mixed with a second PCR mixture containing two fluorescent-labelled specific primers and a non-template directed primer. Five units of FIREPol DNA polymerase (Solis BioDyne, Estonia) were used for each reaction. Reactions were run at the following cycling conditions: 5 min at 94°C, 30 and 35 cycles (for the first and second reactions of the nPCR, respectively) of 30 s at 94°C, 45 s at 45°C and 90 s at 70°C and a final elongation at 70°C for 10 min. The nPCR products were run on a 1.5% agarose gel. Based on relative intensity samples were diluted (1:100, 1:40 and 1:10) and mixed with a GeneScan™ 500 ROX™ dye Size Standard (Applied Biosystems). Samples were air dried overnight and sent to the Genomics Core Laboratory of the Medical Research Council Clinical Science Centre in London for fragment sizing by capillary electrophoresis on an automated sequencer. Highly deionized formamide was added to each sample, and after denaturation, samples were analysed on a 3730xl DNA Analyser (Applied Biosystems Ltd, USA).

### *G6PD* A- and Mediterranean genotyping

Extracted DNA samples were genotyped for SNPs in *G6PD*: 202GA (rs1050828) and *G6PD*:563CT (rs5030868). For the 202GA allele the forward primer was 5′-CTGGCCAAGAAGATCTACCC-3′ and the reverse primer was 5′-GAGAAAACGCAGCAGAGCACAG-3′ [17]. For the 563CT allele the forward primer was 5′-TGATCCTCACTCCCCGAAGA-3′ and the reverse primer was 5′-GCTTGGCCCCACCTCAGCAC-3′ [18]. All primers were from Sigma-Aldrich (Gillingham, UK). Briefly, 5 µl of DNA was amplified in a total reaction volume of 30 µl according to a protocol published by Fanello et al. [17] using the GoTaq Flexi DNA Polymerase (Promega, USA). For 563CT, the annealing temperature of the first cycle of the touchdown PCR was 71.5°C, decreased by 0.5°C for the next 14 cycles and the annealing temperature for the last 24 cycles was 64.5°C. The fragments amplified with the 202GA primers were digested for 4–16 h at 37°C with the restriction enzyme NlaIII (NEBioLabs, USA). The fragments amplified with the 563CT primers were digested overnight at 37°C with the restriction enzyme MboII (NEBioLabs, USA). The digested DNA was analysed with 2.5% MetaPhor Agarose (Lonza, USA) gel electrophoresis.

### Data analysis

Statistical analysis was conducted using STATA 12 (StataCorp, TX, USA). *MSP*-*2* data were analysed using Peak Scanner (Applied Biosystems, CA, USA, version 1.0). Analyses focused on describing prevalence of subpatent infection, multiplicity of infection and risk factors such as G6PD deficiency. Two-sample Wilcoxon rank-sum (Mann–Whitney) test was used to test the differences in continuous variables between the two study sites. Fisher’s Exact tests were used to test for differences in parasite prevalence between populations. The significance level was set at P < 0.05.

## Results

### Socio-demographic characteristics

For SM4, there was a local census report published in 2013 indicating the presence of 432 households with an estimated total population of 1,633 inhabitants. Of these, 156 households and 298 individuals (18.2% of the total population) participated in the study; the mean family size was 4.8 (range: 1–15). In TQ, 78 households were included with a total of 259 participants (20.75% of the total population); the mean family size was 3.7 (range: 1–12). In the SM4 area the average age of participants was 20.2 years (range: 0.6–95 years) and participants of the TQ area had an average age of 14.3 years (range: 0.4–76 years). The male to female ratio was 0.89 and 1.7 for TQ and SM4 sites, respectively (p = 0.10). Study participants in TQ were significantly younger than in SM4 (P < 0.001).

### Malaria prevalence and complexity of *Plasmodium falciparum* infections

No cases of symptomatic malaria defined as axillary temperature ≥ 37.5°C and a positive blood slide or RDT were detected. In the TQ site *P. falciparum* and *P. vivax* prevalence by nPCR was 5.8% (15/257) and 7.4% (19/257), respectively (Table 1), whereas, in the SM4 site infection prevalence was 4.7% (14/298) and 1.7% (5/298) for *P. falciparum* and *P. vivax*, respectively. Only one case of co-infection with both *P. falciparum and P. vivax* was observed in TQ. nPCR was repeated on the positive samples for confirmation. *P. vivax* parasite prevalence was significantly higher at the TQ site (p = 0.001) while *P. falciparum* parasite prevalence was not significantly different between sites (p = 0.57). *Plasmodium falciparum* parasite prevalence (p ≥ 0.13) and *P. vivax* parasite prevalence (p ≥ 0.61) were not significantly associated with age in categories.

**Table 1:** Percentage distribution of participants by age and malaria prevalence

	Age (years)	% of total (n/N)	Microscopy% of positive (n/N)	RDT% of positive (n/N)	nPCR	
					*Pf*% (n/N)	*Pv*% (n/N)
TQ site	≤5	40.5 (104/257)	–	–	7.7 (8/104)	9.6 (10/104)
	6–15	21.4 (55/257)	–	–	3.6 (2/55)	5.5 (3/55)
	>15	38.1 (98/257)	–	–	5.1 (5/98)	6.1 (6/98)
Total					5.8 (15/257)	7.4 (19/257)
SM4 site	≤5	20.5 (61/298)	–	–	9.8 (6/61)	1.6 (1/61)
	6–15	28.9 (86/298)	–	–	3.5 (3/86)	2.3 (2/86)
	>15	50.7 (151/298)	–	–	3.3 (5/151)	1.3 (2/151)
Total					4.7 (14/298)	1.7 (5/298)

*RDT* rapid diagnostic test, *nPCR* nested polymerase chain reaction, *Pf Plasmodium falciparum*, *Pv Plasmodium vivax.* N refers to the total number of participants and n refers to the ones that belong to the classification or positive for the test. ‘–‘ refers to the negative cases.

All samples that were positive for *P. falciparum* were genotyped for the *MSP-2* gene. The success rate of *MSP-2* genotyping, that may be less sensitive than the 18S nPCR [19], was 58.6% (17/29). The total number of detected alleles within *MSP-2* block was four: two different alleles for each of the 3D7 and Fc27 family, 3D7_263 bp and 3D7_330 bp, and Fc27_365 bp and Fc27_424 bp, respectively. The vast majority of infections were single-clone infection by *MSP-2* genotyping (94.1%; 16/17) with only one sample (5.9%) from TQ site having two *MSP-2* alleles.

### *G6PD* A- and mediterranean genotyping

*G6PD* A- (202GA) variant that predominates in sub-Saharan Africa [20] was successfully genotyped in the present study for 553/555 study participants. Of the genotyped individuals, 0/553 carried this A- variant. In addition, the Mediterranean (563CT) variant was tested since previous studies have reported marked differences between regions in *G6PD* mutations [20]. The genotyping was successful for 553/555 participants, also with 0/553 individuals carrying this variant.

## Discussion

In the current study, a substantial number of submicroscopic *P. falciparum* and *P. vivax* infections were detected in two Ethiopian villages while conventional diagnostics did not detect any malaria infections. Genotyping for *P. falciparum MSP-2* gene suggested that most infections were single-clone in nature. No evidence was observed for G6PD deficiency (G6PDd) in the study populations.

The presence of submicroscopic infections in low endemic settings is increasingly well documented [21]. The current study was extreme in the sense that malaria infections were not detected by microscopy despite >5% parasite prevalence by nPCR for both *P. falciparum* and *P. vivax.* Similar results were reported for *P. falciparum* from Solomon Islands [22] where 13 cases were detected by nPCR while only one of them was found positive by microscopy. In line with the findings in the present study, reports from low [23, 24] and high endemic [25] settings in Ethiopia indicated a marked number of nPCR-positive subpatent cases. A recent meta-analysis of PCR surveys suggested that submicroscopic infections form the source of 20–50% of all human-to-mosquito transmissions [5]. This study also indicated the importance of negative controls and rigorous nPCR testing to avoid false positive results. An evaluation of microscopy-confirmed cases in Ethiopia revealed a substantial rate of false positive results, under-reporting of mixed infections and a significant number of species mismatch [26]. In the present study, in order to avoid the possibility of false positive results from the nPCR, all 18S positive results were re-tested. Furthermore, confirmation was obtained with *MSP-2* genotyping that revealed a high proportion of single-clone infections, which is expected in low transmission settings [27]. It is possible that clones have been missed since the assay may miss minority clones and any genotyping assay is affected by parasite sequestration patterns that may cause clones to be undetectable at certain time points [28]. However, clonal complexity is clearly low in this setting.

It is widely acknowledged that asymptomatic individuals that carry microscopically detectable infection often harbour gametocytes and therefore play an active role in ongoing transmission [4]. However, the relative contribution of submicroscopic parasitaemia to transmission is not clearly known in low endemic settings. Evidence is instead indirectly generated from studies in high endemic African settings indicating that the underlying gametocyte prevalence plays a role in defining the infectious reservoir [29]. Thus, for malaria elimination efforts to have a better chance of sustained long-term success, more information is needed about the distribution and infectiousness of the subpatent reservoir. In addition to that, community interventions that target elimination of malaria, such as mass anti-malarial drug administration or mass screening and treatment (MSAT), need to be critically evaluated and tailored into the local context. A recent MSAT campaign that used RDTs for screening has failed in Zanzibar [30] possibly due to the contribution of infections that were not detected by RDTs. Therefore, the high prevalence of asymptomatic subpatent malaria carriage in low endemic or pre-elimination transmission settings may pose challenges for the nationally adopted strategy of malaria elimination in Ethiopia as well as in other low endemic settings.

The other prevailing challenge in Ethiopia, unlike most of Africa, is the high prevalence of *P. vivax*. *Plasmodium vivax* contributes towards 40% of reported malaria cases in Ethiopia next to *P.falciparum* (60%) [31]. In 2011 Ethiopia reported the highest number of *P. vivax* cases globally (665,813) [32]. The coexistence of the two species makes elimination efforts complicated in Ethiopia. Its unique biological characteristics: existence of hypnozoites, production of gametocytes very early in infections and efficient sporogonic development within the mosquito at a large range of temperatures, make *P. vivax* a difficult malaria species to eliminate [33]. There are even concerns that intervention methods might inadvertently favour one species over another resulting in selection for the more transmissible genotypes of the suppressed parasites [34]. Elimination of *P. vivax* will require a different strategy than *P. falciparum.*

Deployment of drugs such as primaquine which is the only licensed drug that is active against the mature transmission stages of *P. falciparum* [35] and which is also the only available drug that can prevent multiple relapses of *P. vivax* [36] is crucial in the elimination efforts that are underway. However, prior understanding of the presence of G6PDd in the target population is required as primaquine administration in G6PDd individuals is associated with a dose-dependent risk of haemolysis [37]. In the Ethiopian population, no molecular information exists to indicate which variants may be responsible for the G6PDd. Few studies reported *G6PD* A- (202GA) frequency estimates considerably lower than those generally found in sub-Saharan Africa [38] ranging from 0 to 1% [39, 40] confirming a recent geostatistical model-based map that predicted a 1.0% prevalence [41]. In agreement with previous reports from neighboring countries [42], the African A- (202GA) and the Mediterranean (563CT) variants revealed no mutations in the present study. G6PDd due to the two most common mutations appears absent or of very low prevalence in the region. In contrast, a recent study reported a 7.3% absence of G6PD enzyme activity in southwest Ethiopia [43] with a significant degree of variation among different ethnic groups that was also reported in other studies [44, 45]. The approaches in the current study may have missed rare variants of *G6PD*. These findings highlight the need for detailed studies on G6PD enzyme activity in combination with G6PD genotyping that may need to look beyond the mutations determined in the current study. The currently available evidence suggests that primaquine may be used in Ethiopia without considerable safety concerns [46]. This is supported by the notion that primaquine was used in Ethiopia for over 25 years up until 1990 [47] with no reports of adverse effects.

## Conclusion

The current report indicates a considerable proportion of submicroscopic *P. falciparum* and *P. vivax* infections in low endemic regions in Ethiopia. The importance of these infections for onward disease transmission is unknown. The apparent absence of G6PDd suggests that primaquine may be used in combination with schizonticidal treatment to clear *P. vivax* hypnozoites and *P. falciparum* transmission stages.

## Abbreviations

G6PD: glucose 6-phosphate dehydrogenase
G6PDd: G6PD deficiency
MSAT: mass screening and treatment
*MSP-2*: merozoite surface protein-2
nPCR: nested polymerase chain reaction
RDT: rapid diagnostics test
SM4: Salayish Mender 4
TQ: Tatta-qirchiqircho
